# Optimizing the Direction and Order of the Motion Unveiled the Ability of Conventional Monolayers of Human Induced Pluripotent Stem Cell-Derived Cardiomyocytes to Show Frequency-Dependent Enhancement of Contraction and Relaxation Motion

**DOI:** 10.3389/fcell.2020.542562

**Published:** 2020-09-10

**Authors:** Hiroko Izumi-Nakaseko, Koki Chiba, Mihoko Hagiwara-Nagasawa, Ayano Satsuka, Ai Goto, Yoshio Nunoi, Ryuichi Kambayashi, Akio Matsumoto, Yoshinori Takei, Yasunari Kanda, Atsuhiko T. Naito, Atsushi Sugiyama

**Affiliations:** ^1^Department of Pharmacology, Faculty of Medicine, Toho University, Tokyo, Japan; ^2^Division of Pharmacology, National Institute of Health Sciences, Kanagawa, Japan; ^3^Department of Aging Pharmacology, Faculty of Medicine, Toho University, Tokyo, Japan; ^4^Department of Translational Research & Cellular Therapeutics, Faculty of Medicine, Toho University, Tokyo, Japan; ^5^Department of Physiology, Division of Cell Physiology, Graduate School of Medicine, Toho University, Tokyo, Japan

**Keywords:** contraction velocity-frequency relationship, frequency-dependent enhancement of relaxation, human induced pluripotent stem cell-derived cardiomyocytes, field potential, motion vector

## Abstract

Contractility of the human heart increases as its beating rate is elevated, so-called positive force-frequency relationship; however, human induced pluripotent stem cell-derived cardiomyocytes (hiPSC-CMs) have been reported to exert a negative force-frequency relationship. We tested the hypothesis that the regulation of motion directions by electrical pacing and/or oxygen supply may improve the electro-mechanical properties of hiPSC-CMs monolayers. To better evaluate the spatial and temporal relationship between electrical excitation and contractile motion, we simultaneously observed the field potential and motion vector of hiPSC-CMs sheets. Under spontaneous contraction, although an electrical excitation originating from a region propagated unidirectionally over the cell sheet, contraction wave started from multiple sites, and relaxation wave was initiated from a geometric center of hiPSC-CMs sheet. During electrical pacing, contraction and relaxation waves were propagated from the stimulated site. Interestingly, the maximum contraction speed was more increased when the hiPSC-CMs sheet was stimulated at an area relaxation initiated under spontaneous condition. Furthermore, motion vector analysis demonstrated that “positive contraction velocity-frequency relationship” in contraction and “frequency-dependent enhancement of relaxation” were produced in the cell sheet by optimizing the direction and order of the contractile motion with pacing at the relaxation-initiating area. A close analysis of motion vectors along with field potential recording demonstrated that relaxation process consists of fast and slow phases, and suggest that intracellular Ca^2+^ dynamics may be closely related to functions of Ca^2+^-ATPase pump and Na^+^-Ca^2+^ exchangers. Namely, the slow relaxation phase occurred after the second peak of field potential, suggesting that the slow phase may be associated with extrusion of Ca^2+^ by Na^+^-Ca^2+^ exchangers during repolarization. Increase of oxygen concentration from 20 to 95% as well as β-adrenergic stimulation with isoproterenol accelerated the fast relaxation, suggesting that it could depend on Ca^2+^ uptake via Ca^2+^-ATPase during the depolarization phase. The ratio of maximum contraction speed to field potential duration was increased by the β-adrenergic stimulation, indicating the elevated contraction efficiency per Ca^2+^-influx. Thus, these findings revealed potential ability of conventional monolayers of hiPSC-CMs, which will help apply them to translational study filling the gap between physiological as well as pharmacological studies and clinical practice.

## Introduction

Positive force-frequency relationship in ventricular muscle has been observed in guinea-pigs, rabbits and humans, which cannot be detected in mice or rats (Kurihara and Sakai, 1985; Pieske et al., 1999; Maier et al., 2000). Enhancement of twitch force occurs along with increasing sarcoplasmic reticulum (SR) Ca^2+^ content in ventricular muscle (Kurihara and Sakai, 1985; Pieske et al., 1999; Maier et al., 2000). In a previous study using the human hearts, non-failing myocardium showed positive force-frequency relationship in twitch force in parallel with an increase of SR Ca^2+^ content, but failing myocardium exerted negative force-frequency relationship in it with decreased SR Ca^2+^ content (Pieske et al., 1999). Therefore, positive force-frequency relationship in contraction can be observed in matured and non-pathological human cardiomyocytes, which makes many researchers focus on the maturation of human induced pluripotent stem cell-derived cardiomyocytes (hiPSC-CMs) to mimic the physiology of the intact human heart. For this purpose, tissue engineering has been applied to facilitate hiPSC-CMs maturation; for example, hiPSC-CMs were mixed in hydrogel solution to prepare 3-dimentional tissue form, which were set on elastic pillars (Ronaldson-Bouchard et al., 2019), on the Biowire II (Feric et al., 2019), or in troughs of Tissue Train 6-well plates (Ruan et al., 2016). After the incubation under static stress and electrical pacing, they obtained the ability showing the positive force-frequency relationship in their twitch force together with changes including rod-shaped morphology and aligned sarcomeres (Ruan et al., 2016; Ronaldson-Bouchard et al., 2019; Feric et al., 2019). Since those experimental systems may be complex to perform, expensive and less versatile, and need hundreds of thousands to millions of hiPSC-CMs per engineered tissue, we adopted the monolayers of hiPSC-CMs which have been used to detect proarrhythmic and anti-arrhythmic potentials of a drug using extracellular field potential recordings (Ando et al., 2017; Izumi-Nakaseko et al., 2017a, b, 2018; Blinova et al., 2018). However, negative force-frequency relationships have been shown in the monolayers of hiPSC-CMs (Sasaki et al., 2018) possibly because of their random sarcomere alignment, the lack of transverse tubule or partially developed Ca^2+^ handling (Yang et al., 2014).

In order to solve such problems in the engineered tissues and conventional monolayers of hiPSC-CMs as described above, we investigated the effects of electrophysiological and biochemical interventions on the conventional monolayers including the regulation of the contraction direction and oxygen tension. While oxygenation of culture medium by air is generally considered to be enough for the cell sheet to keep their basal physiological functions, it is unknown whether the oxygenation by air may be enough for hiPSC-CMs sheets particularly under higher frequency pacing and/or pharmacological β-adrenergic stimulation. In order to develop a method that can regulate the contractile direction, we first prepared mono-layered, high cell-density sheets of hiPSC-CMs set on the probe of the microelectrode-array system which can be paced via a set of two electrodes arbitrarily chosen from 64 electrodes (Izumi-Nakaseko et al., 2017b). Second, we combined microelectrode array measurement and motion vector analysis in order to evaluate the spatial and temporal relationships between electrical excitation and contractile motion propagating over the cell sheet, thus making it possible to study how the regulation of motion directions may improve the mechanophysiological property of hiPSC-CMs sheets. The motion vector analysis with a phase-contrast microscopy enables to observe the multi-directional motion in a cell sheet with high spatiotemporal resolution, which could indirectly provide the information of Ca^2+^ dynamics in each cell, the electrical excitation waveform of sub-cell population, and the cell-to-cell mechanical interaction (Hayakawa et al., 2014; Sugiyama et al., 2019). Since the contraction velocity can be converted to the stretch velocity of the series elastic elements (Hill, 1938; Linke et al., 1999), the elevation of the contraction velocity would be linearly related to the increase of the contractile force in the cell sheet which adheres to the plastic material of the probe. Third, the effect of oxygen tension on the cell motion and field potential was analyzed at control as well as in the presence of 10 nM isoproterenol which would increase the oxygen demand. We propose that such currently used manipulations significantly improve the utility of the conventional monolayers of hiPSC-CMs for simultaneously assessing the inotropic and lusitropic actions of a chemical compound along with its electropharmacological property.

## Materials and Methods

### Culture of hiPSC-CMs Sheets

Human iPSC-CMs (iCell^®^ Cardiomyocytes, FUJIFILM Cellular Dynamics, Inc., Madison, WI, United States) had been incubated for 30∼32 days after differentiation, which were preserved in liquid N_2_ (Ma et al., 2011). The cryopreserved hiPSC-CMs (iCell^®^ Cardiomyocytes^2^; FUJIFILM Cellular Dynamics, Inc.) were purchased and cultured as previously described (Izumi-Nakaseko et al., 2018). A volume of 2 μL of the cell suspension containing 1.5 × 10^4^ cells/μL was plated onto 64-microelectrode array (MED probe; MED-P515A, Alpha MED Scientific Inc., Osaka, Japan) after having coated them with fibronectin. The culture medium (maintenance medium; FUJIFILM Cellular Dynamics, Inc.) around the probe was fully replaced with fresh one once a week. The cardiomyocytes were cultured for 3–5 days to form a cell sheet with spontaneous and synchronous electrical automaticity, which were used for experiments within 3 weeks. The age of hiPSC-CMs at the experiment was calculated as 44∼66 days old on the examination of pacing sites and 66∼70 days old on that with isoproterenol and oxygen concentration.

### Field Potential Recordings and Image Acquisitions

The hiPSC-CMs sheet was incubated in 1 mL of culture medium with 22 mm in diameter and 2.6 mm in depth at 37°C in a stage-top chamber set on the stage of a live cell imaging system, SI8000 (Sony Imaging Products & Solutions Inc., Tokyo, Japan), which was filled and equilibrated for > 30 min at 37°C with gas mixture of 95% air + 5% CO_2_ (normal oxygen supply), or of 95% O_2_ + 5% CO_2_ (high oxygen supply) using a gas controller (INUM-MED-F1, TOKAI HIT Co., Ltd., Shizuoka, Japan). Then, the MED probe was connected to the amplifiers (MED-A64HE1S and MED-A64MD1, Alpha MED Scientific Inc.). The hiPSC-CMs sheet was electrically driven through a pair of neighboring electrodes selected from 64 ones. The stimulation pulses were biphasic, rectangular in shape, 12–50 μA in amplitude (about three times the threshold current) and of 0.4 ms duration, which were applied on the cell sheets in cycle lengths of 600–1,400 ms. In order to examine the effects of pacing sites on the cell sheet, we needed to choose the stimulation sites within 1,050 × 1,050 μm^2^ of the microelectrode array, which was too small to necessarily set four edges. Thus, we selected the one pacing site where the threshold current was the lowest among the edges to reduce the damage of the cell sheets. Isoproterenol of 10 nM was applied to increase the oxygen demand of the cell sheet under each of the oxygen conditions. The rate-adapted field potential duration was assessed under a train of 15 stimuli at a cycle length of 600–1,400 ms before and after the drug treatment. Field potentials of the hiPSC-CMs sheet at 62 microelectrodes were acquired with high- and low-pass filters of 0.1 and 5 kHz, respectively. Field potentials were digitized at a sampling rate of 20 kHz with a MED64-Basic system (Alpha MED Scientific Inc.). Simultaneously, images of the cell sheet motion in the area of 1,365 × 1,365 μm^2^ square were acquired with SI8000 (Sony Imaging Products & Solutions Inc.) at a frame rate of 150 Hz. Image acquisitions and microelectrode array recordings were synchronized using external triggering options of the MED64-Basic system.

### Drugs

(−)-Isoproterenol hydrochloride was purchased from Sigma-Aldrich Japan K.K. (Tokyo, Japan).

### Data Analyses

**FIGURE 1:**
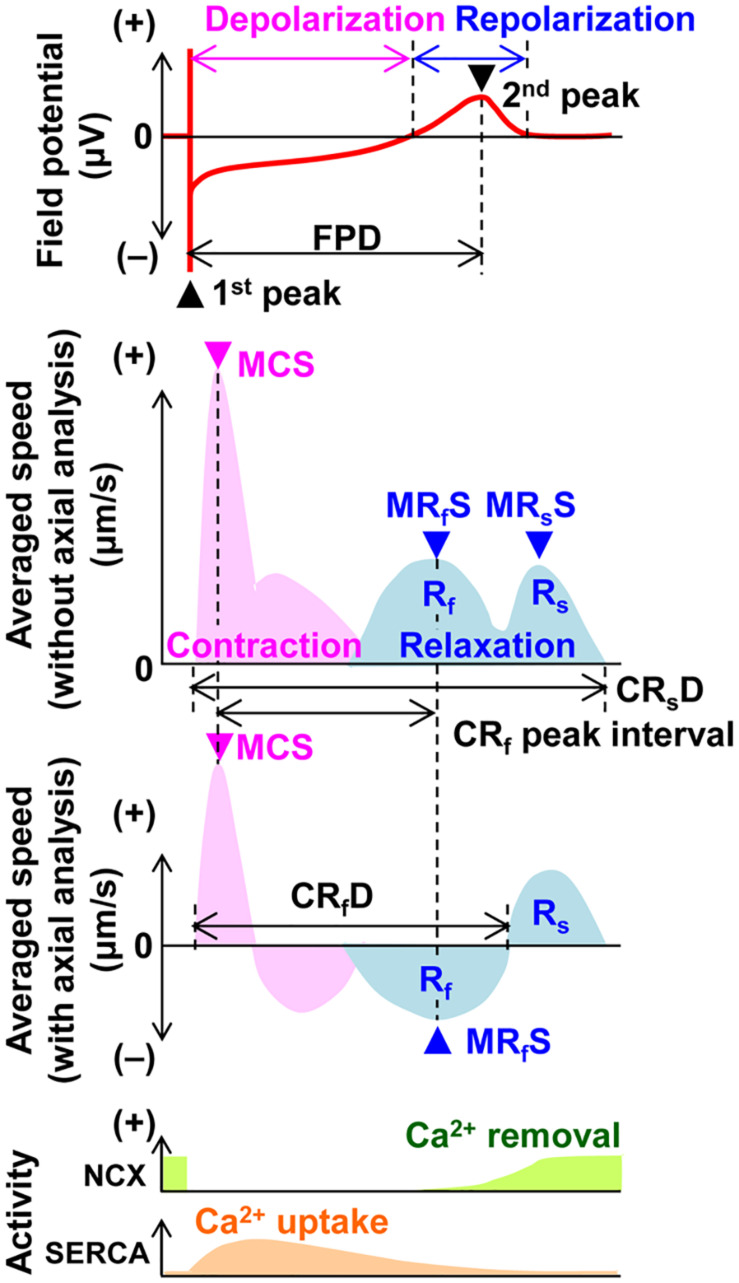
Illustration of the field potential (top panel), motion velocity (middle 2 panels) and postulated activities of Na^+^-Ca^2+^ exchanger (NCX) and SERCA2a (SERCA) (bottom panel) of the human induced pluripotent stem cell-derived cardiomyocytes sheet during a cardiac cycle. The field potential includes the information of extracellular membrane potential of the cells and ion concentration changes by ion channel opening in the space surrounded by cells’ membrane and the surface of the probe. The changes of motion velocity during contraction (pink) and relaxation (blue) are presented without (upper) and with axial analysis (lower), respectively. Activities of Ca^2+^ removal by NCX and Ca^2+^ uptake by SERCA are postulated using the information on the NCX activity and intracellular Ca^2+^ dynamics during an action potential, and Ca^2+^ dependent SERCA activity in the rabbit ventricle (Bers, 1987; Shattock and Bers, 1989; Bassani et al., 1994). FPD, field potential duration; MCS, maximum contraction speed; MR_f_S, maximum fast-relaxation speed; MR_s_S, maximum slow-relaxation speed; CR_s_D, contraction-slow relaxation duration; CR_f_ peak interval, contraction-fast relaxation peak interval; CR_f_D, contraction-fast relaxation duration; R_f_, fast relaxation phase; and R_s_, slow relaxation phase.

Field potential duration and excitation conduction were analyzed with Mobius software (Alpha MED Scientific Inc.) as previously described (Izumi-Nakaseko et al., 2018). Since the field potential duration was largely shortened by the initial several electrical pulses and gradually plateaued during the electrical pacing of 15 pulses, the traces from the 15th electrical stimulation were adopted for analysis. Motion vectors of cell sheet movements were extracted and analyzed by SI8000C Analyzer software (version 1.05.000; Sony Imaging Products & Solutions Inc.) (Hayakawa et al., 2014; Sugiyama et al., 2019). A region of interest (ROI) was set in two ways; (1) a whole observation area of 1,365 × 1,365 μm^2^ square (Large ROI), and (2) multiple 200 × 200 μm^2^ ones (Small ROIs). Contraction velocity of the hiPSC-CMs sheet gradually increased during electrical pacing, and it took about 5 s before the contraction velocity reached the new steady-state level. Based on this observation, motion vectors were analyzed ≥ 5 s after the start of electrical pacing in Large as well as Small ROIs to obtain the stable contractions. Axes for axial analysis of motion vectors in the Small ROIs were set based on the positions toward pacing sites. Figure 1 illustrates the relationship among field potential waveform (top panel) and averaged motion speed changes without and with axial analysis (middle panels), postulated activity of Na^+^-Ca^2+^ exchanger and SERCA2a (bottom panel) with variables used in this study. Although “interval” as well as “duration” means the time interval between two points, we used these two terms in the same manner as described in the previous studies (Hayakawa et al., 2014; Izumi-Nakaseko et al., 2017a, b). Activities of Ca^2+^ removal by Na^+^-Ca^2+^ exchanger and Ca^2+^ uptake by SERCA2a are based on the information on the Na^+^-Ca^2+^ exchanger activity and intracellular Ca^2+^ dynamics during an action potential, and Ca^2+^ dependent SERCA activity in the rabbit ventricle (Bers, 1987; Shattock and Bers, 1989; Bassani et al., 1994). The ratio of maximum contraction speed toward field potential duration (maximum contraction speed/field potential duration) was calculated to estimate the contraction efficiency. To analyze the temporal relationship of the terminal points between repolarization and relaxation, interval was calculated by subtracting the field potential duration from either of the contraction-fast relaxation duration or contraction-slow relaxation duration at each pacing frequency.

Statistical analysis was performed with the software GraphPad Prism 6 (ver 6.03, GraphPad Software, Inc., La Jolla, CA, United States). Statistical significances within a parameter were assessed with one-way, repeated measures analysis of variance (ANOVA) followed by the uncorrected Fisher’s least significant difference test for mean value comparison. Statistical significances before and after the treatment of the drugs were assessed with two-way, repeated measures ANOVA followed by the uncorrected Fisher’s least significant difference test for mean value comparison. Data were presented as mean ± S.E.M. *P*-values < 0.05 were considered to be statistically significant.

## Results

### The Effects of Electrical Pacing on the Field Potential, and the Contraction and Relaxation Processes of hiPSC-CMs Sheets

We prepared one layered, high cell-density sheets of hiPSC-CMs, and simultaneously recorded the field potentials and motion vectors to better understand electro-mechanical relationships in the cell sheets.

**FIGURE 2:**
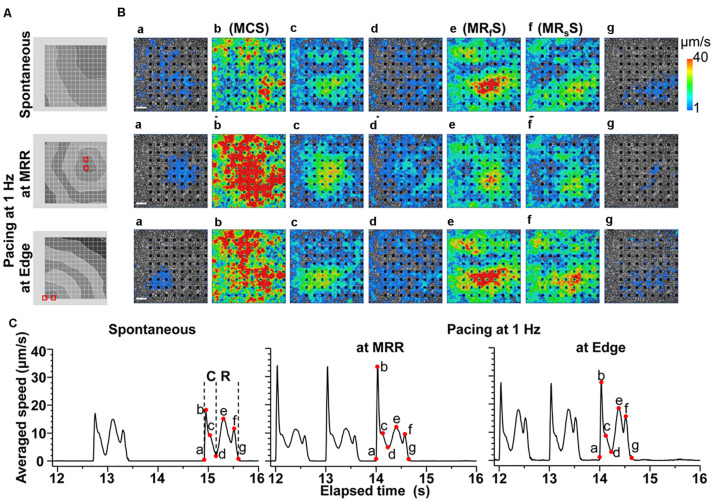
Representative field potentials and motion vectors detected during contraction and relaxation phases of a human induced pluripotent stem cell-derived cardiomyocytes sheet during spontaneous activity, and electrical pacing around maximum relaxation region (MRR) or apart from MRR (Edge) under the mixed gas consisting of 95% O_2_ + 5% CO_2_. **(A)** Activation map of each excitation under spontaneous activity (Spontaneous, top), or under electrical pacing at MRR (middle) or Edge (bottom). Red squares indicate the pacing electrodes. The interval between isochrones is 1 ms. **(B)** The visualized velocity of cell motions on phase-contrast images of the cell sheet during contraction (a∼d) and relaxation (d∼g) under spontaneous activity (Spontaneous, top), or during electrical pacing at MRR (middle) or at Edge (bottom). White bars indicate 200 μm in length. **(C)** The representative traces of averaged speed under spontaneous activity (Spontaneous, left), or during electrical pacing at MRR (middle) or at Edge (right). These traces were obtained from whole observation area of 1,365 × 1,365 μm^2^ square shown in panel B. The last 3 motions under electrical pacing consisting of 15 pulses at 1 Hz are shown (middle and right). Labels of “a” to “g” in panel B correspond to red points with “a” to “g” in panel C during contraction (C) and relaxation (R). Note that b, e and f represent the maximum speeds of contraction (MCS), fast relaxation (MR_f_S), and slow relaxation (MR_s_S), respectively.

A spontaneous electrical excitation from a certain region propagated unidirectionally over the cell sheet (Figure 2A Spontaneous), which evoked contractions with various excitation-contraction intervals, forming a synchronous movement as shown in Figure 2B (Spontaneous a∼b, Supplementary Videos S1, S2). However, the maximal relaxation motion was observed around the center of the cell sheet (Figure 2B; see Spontaneous e∼g, Supplementary Videos S1, S2).

**FIGURE 3:**
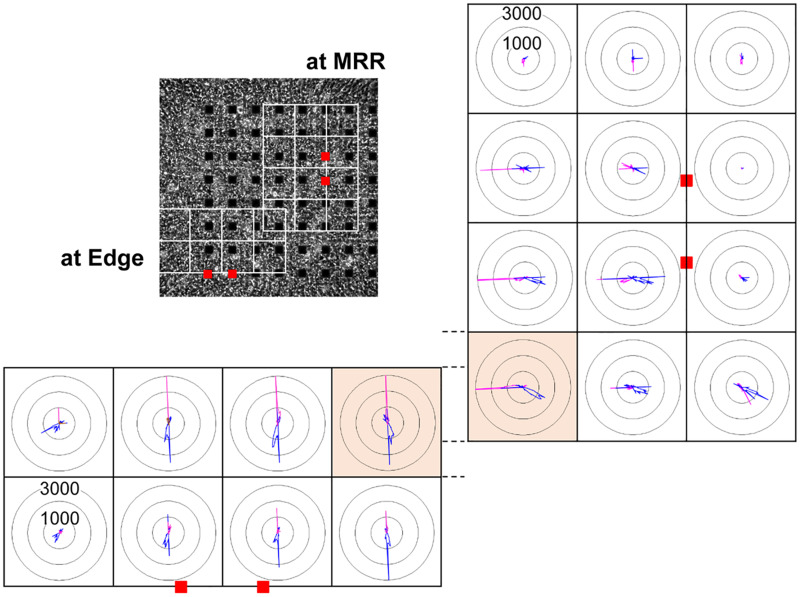
Representative histogram of motion vector direction acquired from each of the small regions of interest (ROIs) consisting of 200 × 200 μm^2^ square (white squares) from the same human induced pluripotent stem cell-derived cardiomyocytes sheet as that in Figure 2, when paced around maximum relaxation region (MRR, right) or apart from MRR (Edge, bottom) under the mixed gas consisting of 95% O_2_ + 5% CO_2_ are shown. Red squares indicate the positions of pacing electrodes on a phase-contrast image of the cell sheet (upper left panel). Each panel indicates the distribution of motion vectors detected during the contraction (pink) and relaxation phases (blue) in 360-degree under electrical pacing. The motion vectors during contraction and relaxation were extracted from the last 9 out of 15 excitations, and were sorted by the direction in every 2.5 degree. The numbers (1000 and 3000) indicate the number of vectors facing to an arbitrary direction. Highlighted small ROIs with bright orange color partially overlapped when paced at MRR and Edge. Note that their patterns of vector direction were quite different.

In order to investigate how the pacing positions can affect the excitation-contraction relationship, we electrically paced the cell sheet via the electrodes near the maximal relaxation region (Figure 2A MRR) or via those apart from MRR (Figure 2A Edge). The evoked excitation propagated uniformly from the pacing positions to the distal areas, whereas the contraction started around the pacing sites. Pacing the sheets around MRR made the starting area of contraction overlapped on MRR. The maximum contraction speed was higher under these pacing conditions than that under spontaneous one (Figures 2B,C, Supplementary Videos S3, S4). The maximum contraction speed was higher when the electrical stimuli were applied around MRR than around Edge (Figure 2C, see points “b”). In addition, there were fast and slow phases in contraction as well as in relaxation as shown in Figure 2C. Analysis with Small ROIs of 200 × 200 μm^2^ square clarified that the vector directions of contraction and relaxation depended on the electrically paced sites as shown in vector direction histogram (Figure 3, Supplementary Videos S5, S6).

**FIGURE 4:**
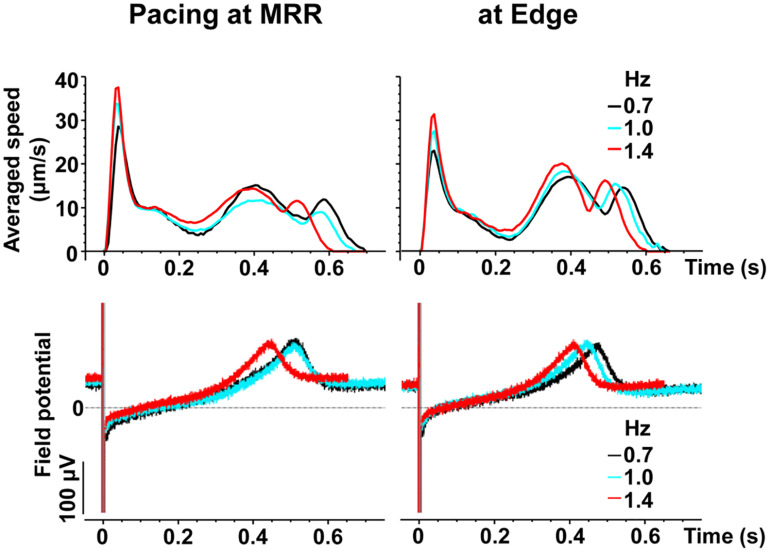
Representative traces of simultaneously obtained averaged speed (upper) and field potential (lower) from the same human induced pluripotent stem cell-derived cardiomyocytes sheet as that in Figure 2, during pacing around maximum relaxation region (MRR, left) or apart from MRR (Edge, right) under the mixed gas consisting of 95% O_2_ + 5% CO_2_. The traces induced by 15^th^ electrical stimulus at frequencies of 0.7 (black), 1 (blue) or 1.4 (red) Hz are superimposed.

**FIGURE 5:**
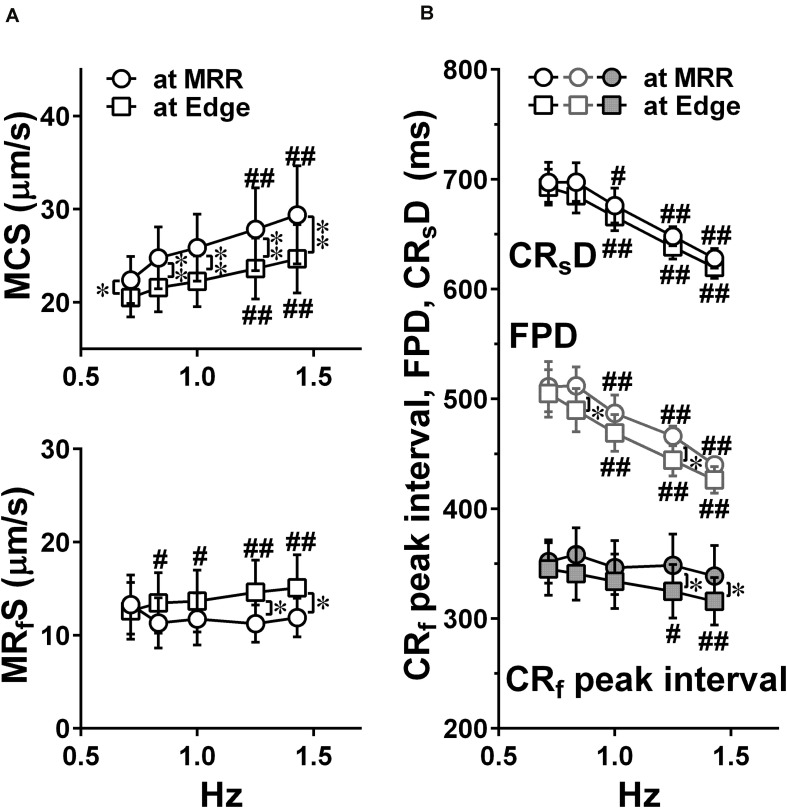
Effects of pacing sites on the relationships between the pacing frequency and either of the maximum contraction speed (MCS, upper) and maximum fast-relaxation speed (MRfS, lower) **(A)**, the contraction-slow relaxation duration (CRsD; interval of “a” to “g” in Figure 2C), field potential duration, and contraction-fast relaxation peak interval (CRf peak interval; interval of “b” to “e” in Figure 2C) **(B)** in human induced pluripotent stem cell-derived cardiomyocytes sheets at the age ≥ 44 days old during pacing at MRR or Edge under the mixed gas consisting of 95% O_2_ + 5% CO_2_. Motion vectors were obtained from whole observation area. Data represent as mean ± S.E.M. (*n* = 5). Significant differences from the corresponding values at the lowest frequency of 0.7 Hz were indicated by ^#^*p* < 0.05 and ^##^*p* < 0.01. Significant differences between the values during pacing at two different sites were shown by ^∗^*p* < 0.05 and ^∗∗^*p* < 0.01.

Representative traces of simultaneously obtained field potential waveform and averaged motion speed from whole observation area of 1,365 × 1,365 μm^2^ square (Large ROI) under electrical pacing at 0.7, 1.0, and 1.4 Hz were overlaid in Figure 4. The relationships between the pacing frequency and either of the maximum contraction speed, maximum fast-relaxation speed, contraction-slow relaxation duration, field potential duration or contraction-fast relaxation peak interval are summarized in Figure 5. The conduction speeds during pacing at 1 Hz around MRR and Edge were calculated to be 0.19 ± 0.01 and 0.23 ± 0.02 m/s, respectively (*p* < 0.05) for the cell sheets of 44∼66 days old. Positive contraction velocity-frequency relationship was demonstrated in the maximum contraction speed which was higher in the pacing condition around MRR than around Edge (Figure 5A upper). The sheet also showed a frequency-dependent elevation of relaxation speed when the pacing was applied around Edge, which was not observed when it was done around MRR (Figure 5A lower). The field potential duration and contraction-slow relaxation duration were shortened in parallel in a frequency-dependent manner (Figure 5B). The field potential duration was shorter when paced around Edge than around MRR. The contraction-fast relaxation peak interval was also shortened in a frequency-dependent manner only when paced around Edge.

### Effects of Oxygen Supply on the Field Potential, and the Contraction and Relaxation Processes of hiPSC-CMs Sheets

**FIGURE 6:**
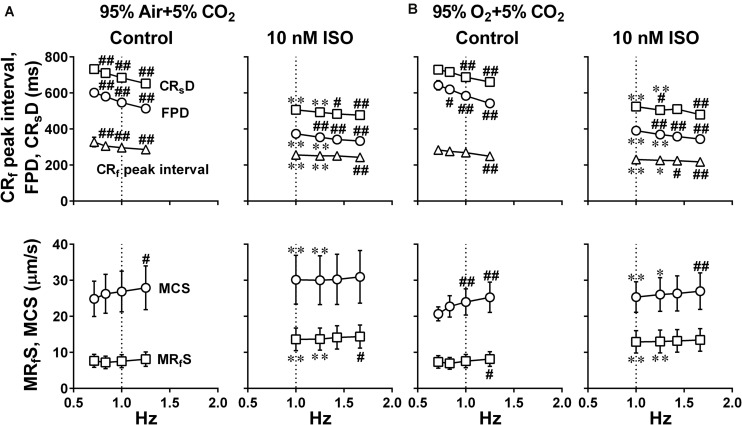
Effects of the difference of oxygen tension; mixed gas of 95% air + 5% CO_2_ **(A)** and 95% O_2_ + 5% CO_2_ **(B)**, on each of the variables of motion vectors and field potential waveforms obtained from the whole observation area (Large ROI) in human induced pluripotent stem cell-derived cardiomyocytes sheets at the age ≥ 66 days old. Summary of the relationships between the pacing frequency and either of the contraction-slow relaxation duration (CR_s_D), field potential duration (FPD), contraction-fast relaxation peak interval (CR_f_ peak interval) (upper panels), maximum contraction speed (MCS) or maximum fast-relaxation speed (MR_f_S) (lower panels) is shown at control (Control) and in the presence of 10 nM isoproterenol (10 nM ISO). Each variable of motion vectors was obtained from whole observation area. Data represent as mean ± S.E.M. (*n* = 5). Significant differences from the corresponding values at the lowest frequency of 0.7 Hz at control and 1 Hz in the presence of 10 nM isoproterenol are indicated by ^#^*p* < 0.05 and ^##^*p* < 0.01. Significant differences between the values at control and in the presence of 10 nM isoproterenol are shown by ^∗^*p* < 0.05 and ^∗∗^*p* < 0.01.

Since it is unknown whether oxygenation of culture medium by air may be enough for the contractility of hiPSC-CMs sheets, we examined whether increasing oxygen supply could enhance the contraction and relaxation at control and in the presence of 10 nM isoproterenol using hiPSC-CMs sheets at the age of 66∼70 days old. As shown in Figure 4, two peaks of relaxation in averaged speed (fast and slow relaxations) were observed before and after the second peak of field potential waveform, respectively in the mixed gas of 95% O_2_ + 5% CO_2_. The relationships between the pacing frequency and either of contraction-slow relaxation duration, field potential duration, contraction-fast relaxation peak interval, maximum contraction speed or maximum fast-relaxation speed extracted from Large ROI under the mixed gas of normal or high oxygen tension were summarized in Figure 6. In both gas conditions, contraction-slow relaxation duration, field potential duration and contraction-fast relaxation peak interval were shortened in a frequency-dependent manner. Ten nM of isoproterenol significantly shortened these parameters compared with those under pre-drug control conditions; and the extent of frequency-dependent shortening in the parameters was diminished but significant in the presence of isoproterenol (Figures 6A,B, upper). Isoproterenol shortened the field potential duration by about 200 ms at the frequency of 1Hz. Data at 0.71 and 0.83 Hz could not be obtained in the presence of isoproterenol because of accelerated spontaneous automaticity rate. Expression level of β_1_ adrenoceptor in hiPSC-CMs at 44 days old of hiPSC-CMs was measured, which was about one third of the adult heart (Supplementary Figure S1). The conduction speed for the sheets of 66∼70 days old during pacing at 1 Hz with normal and high oxygen supply was calculated to be 0.22 ± 0.02 and 0.21 ± 0.03 m/s (*p* = 0.61) in control, and 0.26 ± 0.02 and 0.24 ± 0.02 m/s (*p* = 0.44) after 10 nM isoproterenol treatment, respectively. In both control conditions, the high oxygen supply made the frequency-dependent changes of contraction-slow relaxation duration and contraction-fast relaxation peak interval smaller compared with those under normal oxygen one (Figures 6A,B, Control, upper). As shown in Figures 6A,B, (lower), frequency-dependent increase of maximum contraction speed and maximum fast-relaxation speed was observed in control conditions under normal and high oxygen supply except for the maximum fast-relaxation speed under normal oxygen supply. Isoproterenol increased both of maximum contraction speed and maximum fast-relaxation speed significantly. The positive contraction velocity-frequency relationship in contraction under high oxygen supply was more obvious than that under normal oxygen one.

**FIGURE 7:**
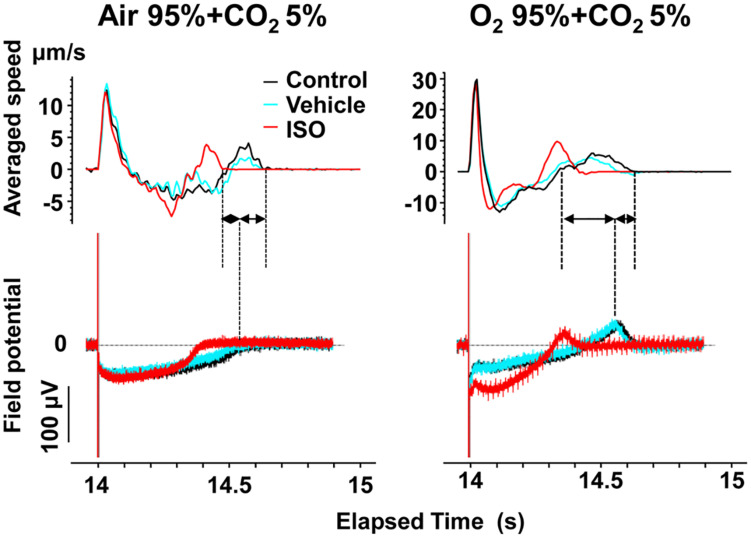
Representative traces of simultaneously recorded averaged speed and field potential acquired from Small ROIs of 200 × 200 μm^2^ of a human induced pluripotent stem cell-derived cardiomyocytes sheet. Traces of averaged speed and field potential induced by 15th electrical stimulus at 1 Hz are shown at control (Control, black), vehicle water (Vehicle; blue) and 10 nM isoproterenol (ISO, red) under the mixed gas conditions consisting of 95% air + 5% CO_2_ (left) or 95% O_2_ + 5% CO_2_ (right). Dashed lines and solid arrows were prepared for the traces at control.

**FIGURE 8:**
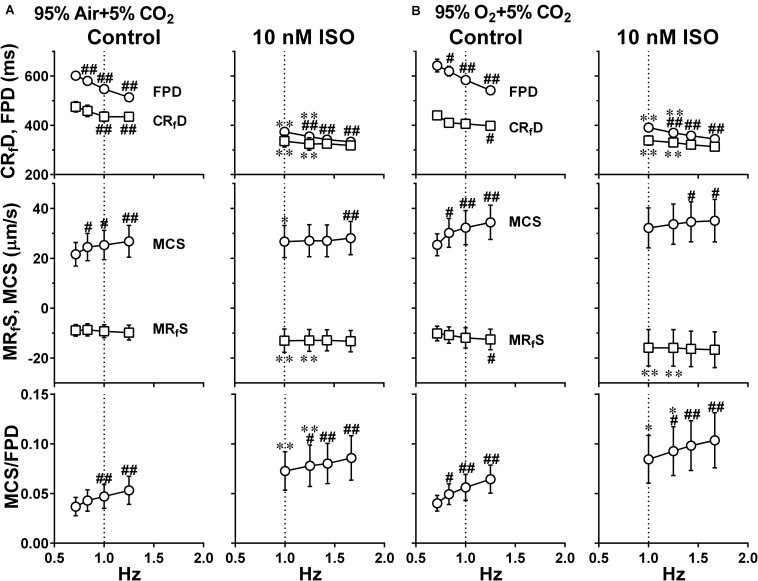
Effects of the difference of oxygen tension; mixed gas of 95% air + 5% CO_2_ **(A)** and 95% O_2_ + 5% CO_2_ **(B)**, on each of the variables of motion vectors and field potential waveforms acquired from Small ROIs of 200 × 200 μm^2^. This data was obtained from the same human induced pluripotent stem cell-derived cardiomyocytes sheets at the age ≥ 66 days old as those used for obtaining the data shown in Figure 6. Summary of the relationships between the pacing frequency and either of the field potential duration (FPD), contraction-faster relaxation duration (CR_f_D) (top panels), maximum contraction speed (MCS), maximum fast-relaxation speed (MR_f_S) (middle panels) or maximum contraction speed/field potential duration (MCS/FPD) (bottom panels) is shown at control (Control) and in the presence of 10 nM isoproterenol (10 nM ISO). Data represent as mean ± S.E.M. (*n* = 5). Significant differences from the corresponding values at the lowest frequency of 0.7 Hz (Control) or 1 Hz (ISO) are indicated by ^#^*p* < 0.05 and ^##^*p* < 0.01. Significant differences between the values at control and in the presence of 10 nM isoproterenol are shown by ^∗^*p* < 0.05 and ^∗∗^*p* < 0.01.

**FIGURE 9:**
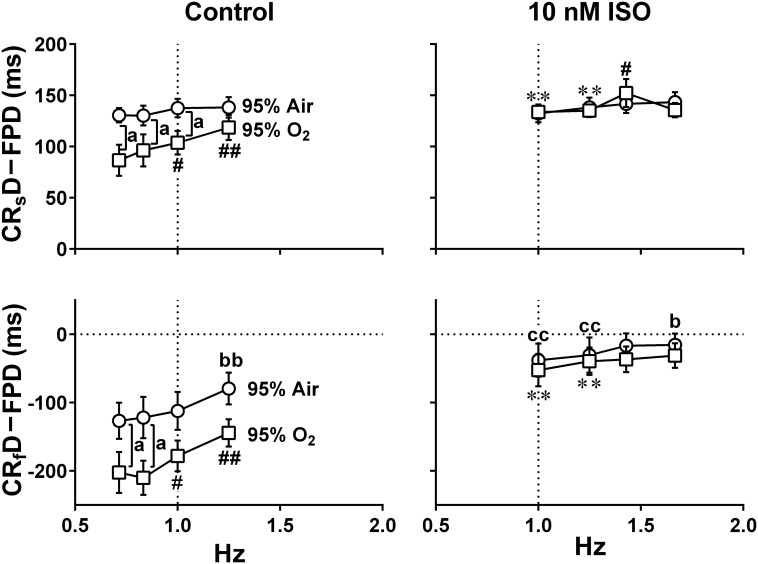
The relationships between the pacing frequency and either of the temporal intervals obtained by subtracting the field potential duration from either of the contraction-slow relaxation duration (upper, CR_s_D–FPD) or the contraction-fast relaxation duration (lower, CR_f_D–FPD) are shown under the mixed gas conditions consisting of 95% air + 5% CO_2_ (95% Air, circles) or 95% O_2_ + 5% CO_2_ (95% O_2_, squares) at control (Control) or in the presence of 10 nM isoproterenol (10 nM ISO). The values of CR_s_D–FPD and CR_f_D–FPD were calculated from the data shown in Figures 6, 8. Note that early-onset of the relaxation phase was induced by high oxygen supply. Data represent as mean ± S.E.M. (n = 5). Significant differences between the values under normal and high oxygen tensions are depicted by ^a^*p* < 0.05. Significant differences from the corresponding values at the lowest pacing frequency of 0.7 Hz (Control) or 1 Hz (ISO) are indicated by ^b^*p* < 0.05 and ^bb^*p* < 0.01 for 95% Air and by ^#^*p* < 0.05 and ^##^*p* < 0.01 for 95% O_2_. Significant differences between the values at control and in the presence of 10 nM isoproterenol are shown by ^cc^*p* < 0.01 for 95% Air and by ^∗∗^*p* < 0.01 for 95% O_2_.

To better analyze the fast relaxation phase, we adopted the axial analysis of motion vectors using Small ROIs (Figure 1 middle). Representative traces of averaged speed and field potential waveforms recorded at control and in the presence of vehicle as well as 10 nM of isoproterenol under normal and high oxygen supply are shown in Figure 7, whereas the relationships between the pacing frequency and either of the field potential duration, contraction-fast relaxation duration, maximum contraction speed, maximum fast-relaxation speed, or maximum contraction speed/field potential duration under normal and high oxygen supply are summarized in Figure 8. The value by subtracting field potential duration from either of contraction-slow relaxation duration or contraction-fast relaxation duration is summarized at control (Figure 9, left) and in the presence of isoproterenol (Figure 9, right) under normal and high oxygen supply. The contraction-fast repolarization duration was shorter than the field potential duration in both gas conditions in control (Figures 8A,B, top), whereas high oxygen supply made the end point of fast relaxation phase occur much earlier from the 2nd peak of field potential than that under normal one (Figure 9 lower left). Similarly, high oxygen supply made the end point of slow relaxation phase occur earlier behind the second peak of field potential than that under normal one (Figure 9 upper left). The early-onset of the relaxation phase under high oxygen supply was declined by isoproterenol treatment (Figure 9, right). The positive contraction velocity-frequency relationship in maximum contraction speeds was observed under normal and high oxygen supply in the Small ROI analysis like Large ROI analysis at control (Figure 8, middle); moreover, the high oxygen supply enhanced the relationship. **T**he contraction efficiency (maximum contraction speed/field potential duration) was increased by isoproterenol under normal and high oxygen supply (Figure 8, bottom). Meanwhile, isoproterenol elevated the maximum contraction speed in Large ROIs analysis at 1.00 and 1.25 Hz (Figure 6), which was diminished in Small ROIs analysis (Figure 8). Frequency-dependent enhancement of fast relaxation speed was observed under high oxygen supply in control (Figure 8B, left middle), which was not observed in the other conditions. Maximum fast-relaxation speed was increased by isoproterenol under normal and high oxygen supply (Figures 8A,B, middle).

## Discussion

We demonstrated that a spontaneous electrical excitation originating from some region propagated over the cell sheet and formed a synchronous movement, but the initiation of mechanical contractions did not necessarily accord with the order of electrical excitations in the cell sheet of hiPSC-CMs (Figure 2B, “b” top). These findings indicate that the disorganized contractions would make the net contraction movement less great, and suggest that there might be large variability of the available Ca^2+^ store in SR among the cells during slow automaticity. In addition, relaxation speed was high in the central area and the relaxation movement was propagated from central to peripheral within the cell sheets (Figure 2B, “e” top), indicating that Ca^2+^ uptake rate to SR may be the fastest in the central. This finding also suggests that the central region may be passively stretched.

Next, we electrically paced the cell sheets in order to facilitate Ca^2+^-loading to SR by increasing the excitation rate. Our results demonstrate that the contraction originated from the electrically stimulated regions during pacing (Figure 2B, “a” middle and bottom), and that the direction of contraction and relaxation motions varied depending on the paced sites (Figure 3). The conduction speed paced at 1 Hz was 0.19∼0.23 m/s for the cell sheets of 44∼66 days old (Figure 2A), which was lower limit of the intact human ventricle (0.2–0.3 m/sec) (Levy and Pappano, 2007). Moreover, when electrical pacing was applied at MRR, elevating the pacing frequency from 0.7 to 1.4 Hz increased the maximum contraction speed by + 27% (p < 0.05) (+ 39%/Hz) (Figures 4, 5), indicating a positive contraction velocity-frequency relationship, which has not been reported before in conventional monolayers of hiPSC-CMs. Since the contraction velocity can be converted to the stretch velocity of the series elastic elements (Hill, 1938; Linke et al., 1999), the elevation of the contraction velocity would be linearly related to the increase of the contractile force in the cell sheet which adheres to the plastic material of the probe. Twitch force of human cardiac muscle strip preparation was shown to be linearly increased by + 71% when the stimulation frequency was changed from 0.25 to 3 Hz (+ 26%/Hz) (Pieske et al., 1999). Meanwhile, increase of atrial rate from spontaneous sinus automaticity (74 ± 8 bpm; 1.23 Hz) to that plus 25 bpm (102 ± 15 bpm; 1.7 Hz) and to that plus 50 bpm (134 ± 12 bpm; 2.23 Hz) was reported to linearly increase the left ventricular peak-positive dP/dt of the human *in situ* heart by + 19.4% and + 35.4%, respectively (+ 35.4%/Hz) (Feldman et al., 1988). Thus, the positive contraction velocity-frequency relationship of the contraction speed in the cell sheets could mimic positive force-frequency relationship shown in the human heart. In addition, when the pacing frequency was changed from 0.7 Hz to 1.4 Hz during pacing at Edge, the maximal contraction speed was less elevated by + 18% (*p* < 0.05) (+ 26%/Hz) than that during pacing at MRR (Figure 5A). Electrical excitation was propagated uniformly from electrically paced sites (Figure 2A), suggesting homogeneous distribution of connesxins throughout the cell sheet. Therefore, the connexin would have played a minor role in the observed differences induced by the change of pacing sites. Thus, regulation of the contractile direction by local electrical pacing could be critical to produce the positive contraction velocity-frequency relationship in the monolayers of hiPSC-CMs.

During electrical pacing at Edge, the maximum fast-relaxation speed was elevated (Figure 5A lower) in a frequency-dependent manner. Also, the contraction-slow relaxation duration and contraction-fast relaxation peak interval as well as field potential duration were shortened in a frequency-dependent manner except for contraction-fast relaxation peak interval paced at MRR (Figure 5B). Thus, by electrical pacing at Edge, we could induce the frequency-dependent enhancement of fast-relaxation speed along with early-onset of the fast-relaxation phase in the conventional monolayers of hiPSC-CMs. These results suggest that such a mechanism might be operated in the cell sheet that depolarization frequency-dependent Ca^2+^/calmodulin-dependent kinase II (CaMKII) activation can enhance SERCA2a function via phosphorylation of phospholamban (Braun and Schulman, 1995; De Koninck and Schulman, 1998).

The slow relaxation phase occurred after the 2nd peak of field potential (Figure 4), suggesting that phase 3 repolarization may be associated with the slow relaxation. Since extrusion of Ca^2+^ by Na^+^-Ca^2+^ exchangers is promoted as repolarization proceeds (Kimura et al., 1987; Bers, 2001; Figure 1 bottom), the activity of Na^+^-Ca^2+^ exchangers may partly determine the duration and termination of the slow relaxation phase.

When paced at Edge, the contraction-fast relaxation peak interval (Figure 5B) was shortened and the maximum fast-relaxation speed (Figure 5A lower) was elevated in a frequency-dependent manner, which was not observed when paced at MRR. The fast relaxation would occur during plateau phase of action potential (Figure 4), indicating that the fast relaxation may be induced by Ca^2+^ uptake via Ca^2+^-ATPase, SERCA2a and/or plasma membrane Ca^2+^-ATPase 4 (PMCA4). Since the maximum contraction speed was higher during pacing at MRR than at Edge (Figure 5A upper), basal SERCA2a activity could be higher around MRR than Edge, which could be explained by a previous report that cell-stretch can enhance SERCA2a activity by activating protein kinase G Iα that phosphorylates phospholamban (Scotcher et al., 2016). Therefore, it is possible that stretch-enhanced SERCA2a activity in MRR could enhance the frequency-dependent increase of Ca^2+^ storage and Ca^2+^ release, which might have partly attenuated the relaxation motion despite of enhanced SERCA2a activity through depolarization frequency-dependent activation of CaMKII (Braun and Schulman, 1995; De Koninck and Schulman, 1998).

Air contains 20.93% of oxygen. When the culture medium is equilibrated with mixed gas consisting of 95% air + 5% CO_2_ or 95% O_2_ + 5% CO_2_, their dissolved oxygen concentration is calculated to be 0.004 or 0.021 mL/mL H_2_O at 37°C, respectively, by using the Henry’s law and Bunsen’s absorption coefficient, giving the ratio of high/normal oxygen tension of 4.7. We adopted axial analysis on Small ROIs to examine the temporal relationships between the field potential and cell movement. There was no difference in the maximum contraction and fast-relaxation speeds at 0.7 Hz between both oxygen conditions at control (Control in Figure 6 lower and Figure 8 middle). Higher oxygen supply made the contraction velocity-frequency relationship and frequency-dependent elevation of fast-relaxation speed steeper, indicating that higher oxygen supply may have enhanced SERCA2a activity. Isoproterenol elevated the maximum contraction speed compared with control in the both oxygen conditions. Expression level of β_1_ adrenoceptor in hiPSC-CMs at 44 days old was about one third of the adult heart (Supplementary Figure S1), which is in accordance with those described in a previous report (Wu et al., 2015), suggesting that β_1_ adrenoceptor in the hiPSC-CMs at 66∼70 days old would actually contribute to the observed changes including the elevation of contraction and relaxation speeds by isoproterenol. However, its positive contraction velocity-frequency relationship was diminished or attenuated by isoproterenol (Figure 6 lower and Figure 8 middle). Furthermore, marked abbreviation of field potential duration was induced by isoproterenol (Figures 6, 8, top), which would have decreased Ca^2+^ influx via L-type Ca^2+^ channels. In order to better evaluate the net effect of isoproterenol on contraction, we estimated the contraction efficiency by calculating the ratio of the maximum contraction speed to field potential duration, which unveiled the significant positive contraction velocity-frequency relationship under isoproterenol treatment (Figure 8 bottom). When the positive contraction velocity-frequency relationship in the contraction efficiency was compared between control and isoproterenol conditions, higher oxygen supply made the relationship steeper.

The value of CR_s_D–FPD under normal oxygen supply was not affected by the pacing frequencies, which was also observed with the presence of isoproterenol (Figure 9 upper). Namely, there may be a strong causal relationship between the 2nd peak of field potential and the end point of the slow relaxation, suggesting that recovery of intracellular Ca^2+^ might be highly regulated by Na^+^-Ca^2+^ exchangers which are up-regulated at resting potential (Kimura et al., 1987; Bers, 2001; Figure 1 bottom). Meanwhile, fast- and slow-relaxation terminated earlier with higher oxygen supply at control (Figure 7 right; Figure 9 left), indicating that higher oxygen supply could have improved SERCA2a activity (Figure 1 bottom). However, CR_s_D–FPD and CR_f_D–FPD were gradually prolonged and shortened, respectively, by increasing the pacing frequency, and isoproterenol diminished the difference between the oxygen conditions (Figure 8), indicating that enhanced Ca^2+^-mobilization would have delayed the relaxation.

We used a monolayer sheet consisting of 30,000 hiPSC-CMs which had been spontaneously excited and contracted until the experiments. Miniaturized cardiac tissue consisting of 2,000 hiPSC-CMs was reported to lack the positive force-frequency relationship (Huebsch et al., 2016). Meanwhile, it has been shown that engineered cardiac tissues exerted the positive force-frequency relationships in the twitch forces, but that they consisted of hundreds of thousands to millions of hiPSC-CMs and required static stress and/or frequency-escalated electrical pacing to facilitate their maturation (Ruan et al., 2016; Feric et al., 2019; Ronaldson-Bouchard et al., 2019). Thus, our protocol along with the monolayer sheets can be simple, less expensive, easy-to-construct and versatile, which could be used to study the physiology and pathophysiology of positive contraction velocity-frequency relationship in 2D model systems.

## Limitation

First, since we prepared the cell sheet by dropping a 2 μL of cell suspension onto the microelectrode array in the probe, the cell density in the central area of the cell sheet might be greater than those in peripheral ones. The surface area of the cell sheets was > 2 times greater than that of the observation window of 1,365 × 1,365 μm^2^, possibly reducing the dispersion of the cell density within the window. The window included the central region of the cell sheet, where the 2nd peak amplitude of the field potential was higher, enabling to observe the area having the highest cell density within each cell sheet. Second, the number of experimental trials may not be sufficient for more reliable results with the variability associated with measurements in hiPSC-CMs; however, other researchers as well as we have provided the similar values in variables of field potential and motion vector to those in the current study using the cell sheets by 5∼7 trials (Hayakawa et al., 2014; Nakamura et al., 2014; Uesugi et al., 2014; Ando et al., 2017; Izumi-Nakaseko et al., 2017a, b; Isobe et al., 2018; Sugiyama et al., 2019). Thus, reliable results could be obtained by 5 experimental trials, although increasing the number of trials may further improve the reliability. Third, we used a single cell line that was commercially available, providing some possibility that the observations could be limited to this cell line. Although the cell line we adopted is one of the most widely used cell strains, other cell lines should be tested to verify our observations.

## Conclusion

We demonstrated that regulation of the motion directions in the conventional hiPSC-CMs monolayers enables to produce both “positive contraction velocity-frequency relationship” in contraction and “frequency-dependent enhancement of relaxation”, which will become more obvious by increasing oxygen supply. We also clarified the spatial and temporal relationships between the electrical excitation and biphasic relaxation. Our findings might help interpret the drug-induced inotropic and lusitropic actions detected in hiPSC-CMs monolayers. Thus, currently adopted manipulation revealed potential ability of conventional monolayers of hiPSC-CMs, which will help apply them to translational study filling the gap between basic research and clinical practice.

## Data Availability Statement

All datasets generated for this study are included in the article/Supplementary Material.

## Author Contributions

HI-N and ASu supervised the project and designed the experiments. HI-N and ASa performed and analyzed the experiments. KC, MH-N, AG, YN, RK, AM, YT, YK, and AN provided technical support and discussions. HI-N and ASu wrote the manuscript. All authors reviewed the manuscript.

## Conflict of Interest

The authors declare that the research was conducted in the absence of any commercial or financial relationships that could be construed as a potential conflict of interest.
