# Supplementary material for: Optimizing the Direction and Order of the Motion Unveiled the Ability of Conventional Monolayers of Human Induced Pluripotent Stem Cell-Derived Cardiomyocytes to Show Frequency-Dependent Enhancement of Contraction and Relaxation Motion
Source: Front Cell Dev Biol. 2020 Sep 10;8:542562. doi: 10.3389/fcell.2020.542562 (PMC7511828; doi:10.3389/fcell.2020.542562)
Supplement: Supplementary file 1 [file Data_Sheet_1.docx]

**Supplementary Material**

**Table of Contents**

Supplemental information, Video legends, Table, Figure and Videos

Information S1: Quantitative PCR assays.

Video legend S1: Spontaneous cardiac cycle

Video legend S2: Spontaneous cardiac cycle (heat map)

Video legend S3: Pacing at MRR (heat map)

Video legend S4: Pacing at Edge (heat map)

Video legend S5: Pacing at MRR

Video legend S6: Pacing at Edge

Table S1: List of primer sequences for quantitative PCR assays.

Figure S1: Normalized mRNA expression levels of hADRB1 and hADRB2.

Video S1: Spontaneous cardiac cycle

Video S2: Spontaneous cardiac cycle (heat map)

Video S3: Pacing at MRR (heat map)

Video S4: Pacing at Edge (heat map)

Video S5: Pacing at MRR

Video S6: Pacing at Edge

**Information S1: Quantitative PCR assays**

Total RNA was isolated from iCell^®^ Cardiomyocytes of the age of 44 days old from differentiation using TRIzol reagent (Life Technologies Life Technologies, Carlsbad, CA, USA) and treated with recombinant DNase I (RNase-free) (Takara Bio Inc., Shiga, Japan) to remove residual genomic DNA. Human fetal heart total RNA (Takara Bio Inc.) and human heart total RNA (Takara Bio Inc.) were used for comparative analysis. Quantitative real-time reverse transcription (RT)-PCR of hADRB1, hADRB2 and hGAPDH was performed with a QuantiTect SYBR Green RT-PCR Kit (Qiagen, Valencia, CA, USA) on a QuantStudio 7 Flex real time PCR system (Applied Biosystems, Foster City, CA, USA), as previously reported (Yamada and Kanda., 2019). Relative changes in transcript levels were normalized to mRNA levels of glyceraldehyde-3-phosphate dehydrogenase (GAPDH). Primer sequences used for real-time PCR analysis are shown in Table 1.

**Reference**

Yamada, S., Kanda, Y. (2019). Retinoic Acid Promotes Barrier Functions in Human iPSC-derived Intestinal Epithelial Monolayers. *J Pharmacol Sci.* 140, 337-344. doi:10.1016/j.jphs.2019.06.012.

**Video legend S1** Spontaneous cardiac cycle. Spontaneous cardiac cycles during 10 s within 1,365×1,365 μm^2^ are captured at magnification of ×4 in phase-contrast. The mp4 movie is obtained from the same database used for Figure 2.

**Video legend S2** Spontaneous cardiac cycle (heat map). Motion vectors during one spontaneous cardiac cycle were extracted from the phase contrast video of the same cell sheet as shown in Video S1 and Figures 2B (Spontaneous), and the motion speeds at arbitrary points are indicated using the heat map. The video has information of both phase contrast images and heat map. The speed of video is 0.2 times of the actual.

**Video legend S3** Pacing at MRR (heat map). Motion vectors during one cardiac cycle induced by pacing at MRR were extracted from the phase contrast video of the same cell sheet as shown in Figures 2B (MRR), and the motion speeds at arbitrary points are indicated using the heat map. The video has information of both phase contrast images and heat map. The speed of video is 0.2 times of the actual.

**Video legend S4** Pacing at Edge (heat map). Motion vectors during one cardiac cycle induced by pacing at Edge were extracted from the phase contrast video of the same cell sheet as shown in Figures 2B (Edge), and the motion speeds at arbitrary points are indicated using the heat map. The video has information of both phase contrast images and heat map. The speed of video is 0.2 times of the actual.

**Video legend S5** Pacing at MRR. The last 5 beats in 15 cardiac cycles when paced at 1 Hz at MRR were shown, which were obtained from the same cell sheet as shown in Video S3 and Figure 2B (MRR). The mp4 movie is obtained from the same database used for Figure 2 (MRR).

**Video legend S6** Pacing at Edge. The last 5 beats in 15 cardiac cycles when paced at 1 Hz at MRR were shown, which were obtained from the same cell sheet as shown in Video S4 and Figure 2B (Edge). The mp4 movie is obtained from the same database used for Figure 2 (Edge).

**Table S1** List of primer sequences for quantitative PCR assays

| gene | forward | reverse |
| --- | --- | --- |
| hADRB1 | ATCGAGACCCTGTGTGTCATT | GTAGAAGGAGACTACGGACGAG |
| hADRB2 | TACCAGAGCCTGCTGACCAAGA | AGTCACAGCAGGTCTCATTGGC |
| hGAPDH | GTCTCCTCTGACTTCAACAGCG | ACCACCCTGTTGCTGTAGCCAA |
